# The Contribution of Spatial and Temporal Molecular Networks in the Induction of Long-term Memory and Its Underlying Synaptic Plasticity

**DOI:** 10.3934/Neuroscience.2016.3.356

**Published:** 2016-10-22

**Authors:** Anastasios A. Mirisis, Anamaria Alexandrescu, Thomas J. Carew, Ashley M. Kopec

**Affiliations:** 1 Center for Neural Science, New York University, New York, NY, USA; 2 Department of Biology, New York University, New York, NY, USA; 3 Department of Neuroscience and Physiology, New York University School of Medicine, New York, NY, USA; 4 Department of Pediatrics, Harvard Medical School, Boston, MA, USA

**Keywords:** spatial network, temporal network, learning, memory, growth factors, MAPK, PKA, integration, coincidence detection

## Abstract

The ability to form long-lasting memories is critical to survival and thus is highly conserved across the animal kingdom. By virtue of its complexity, this same ability is vulnerable to disruption by a wide variety of neuronal traumas and pathologies. To identify effective therapies with which to treat memory disorders, it is critical to have a clear understanding of the cellular and molecular mechanisms which subserve normal learning and memory. A significant challenge to achieving this level of understanding is posed by the wide range of distinct temporal and spatial profiles of molecular signaling induced by learning-related stimuli. In this review we propose that a useful framework within which to address this challenge is to view the molecular foundation of long-lasting plasticity as composed of unique spatial and temporal molecular networks that mediate signaling both within neurons (such as via kinase signaling) as well as between neurons (such as via growth factor signaling). We propose that evaluating how cells integrate and interpret these concurrent and interacting molecular networks has the potential to significantly advance our understanding of the mechanisms underlying learning and memory formation.

## 1. Introduction

The ability to form long-lasting memories is an evolutionarily conserved phenomenon that is critical to survival, and the dynamic process of acquiring and storing memories is often compromised in psychiatric and neurodegenerative disorders. It is thus important to understand how memory formation occurs in healthy individuals, and how these processes go awry in pathological states.

Memory can exist in at least three temporal domains best distinguished by the molecular mechanisms engaged in their induction and maintenance. Short-term memory (STM), which lasts on the order of minutes, is mediated by post-translational modifications [1,2]. Intermediate-term memory, which can last several hours, requires protein synthesis but not transcription [3,4], and long-term memory (LTM), which can last anywhere from days to a lifetime, requires both protein synthesis and *de novo* gene expression [1,5]. The study of long-lasting plasticity underlying memory formation has served as a valuable platform for the analysis of several novel molecular mechanisms, including long-term DNA modification [6] and reconsolidation [7]. However, the same complexity that yields such richness and depth in molecular signaling also poses a significant challenge to understanding the process of memory formation. One challenge lies in the temporal profile of LTM: LTMs are induced, consolidated, and expressed over a wide range of temporal scales, resulting in a virtually unlimited number of time points for assessing the molecular and cellular substrates involved. A second challenge lies in the spatial regulation required by the molecular mechanisms underlying LTMs. Specifically, LTM uniquely requires new gene expression, which raises the critical question as to how the synapse, where neural communication initially occurs, and the cell body, where transcription occurs, effectively communicate.

In this review, we will first introduce the concept of *spatial and temporal molecular networks* in the context of learning and memory by reviewing the spatial and temporal profiles of selected molecular signaling cascades critical for long-lasting plasticity and LTM formation. We will then focus on one specific aspect required for memory formation, growth factor signaling, which effectively captures these concepts. Finally, we will discuss how neurons might integrate and interpret these concurrent and interacting molecular networks, and how this overall framework can inform the study of LTM function and dysfunction.

## 2. Spatially- and Temporally-regulated Molecular Signaling in Long-lasting Plasticity and Memory Formation

It is widely appreciated that learning-related stimuli induce molecular signaling cascades (e.g. kinase activation, translation, or transcription) in discrete temporal and spatial profiles dictated by the nature of the stimulus, and these profiles are of critical importance to the resulting functional outcome. In one interesting example, Pineda and colleagues [8] demonstrated that enhancing adenylyl cyclase activity by genetically disrupting the G_i_-coupled receptor that inhibits it, resulted in enhanced long-term potentiation (LTP) in the hippocampus, but this same manipulation actually impaired hippocampal-dependent memory. These data demonstrate that (i) LTP, often considered to be a cellular correlate of memory, is not always an accurate predictor of behavior, and (ii) signaling enhancement above the physiological levels induced by a learning event does not necessarily mean the memory formed will be superior. Although adenylyl cyclase activity, and cyclic adenosine monophosphate (cAMP) signaling in general, is required for memory formation (see discussion below), it is likely to be required at very specific levels and for very discrete periods of time. In the paragraphs that follow we will (i) provide a brief history of the general spatial properties of molecular signaling induced by learning-related stimuli, (ii) review the temporal and spatial profiles of two specific families of kinases critical for long-lasting plasticity and memory: MAPK and PKA, and (iii) highlight examples of the rich interactions between these kinases for molecular and cellular outcomes critical for plasticity.

### 2.1. Spatial properties of neural communication

Convincing evidence of the subcellular compartmentalization of distinct molecular cascades was supported by the notion that long-term synaptic strengthening induced at one synaptic site can facilitate the induction of long-term changes at another site [9,10]. Frey and Morris [9] proposed the critical idea of “synaptic tagging”, that is, a protein synthesis-independent process that sequesters proteins necessary to establish late-LTP at specific synaptic sites. For example, repeated stimulation to discrete synaptic inputs in the CA1 region of hippocampal slices has been demonstrated to induce protein synthesis and late-LTP at those inputs [9]. Subsequently, when weak stimulation, or repeated stimulation in the presence of protein synthesis inhibitors (which precludes the establishment of late-LTP), was applied to a neighboring synaptic input, late-LTP is also established at this input.

The same year, Martin and colleagues proposed in a series of experiments [11] a similar concept: “synaptic capture.” They used the model system *Aplysia* to investigate branch-specific protein synthesis using a bifurcated sensory-motor neuron (SN-MN) co-culture, in which two MNs are synaptically coupled to different branches of a single SN. If one set of SN-MN synapses was exposed to repeated applications of serotonin, long-term synaptic facilitation (LTF) and growth of new synaptic connections occurred in the branch which received stimulation, while the unstimulated branch remained unchanged [11]. Importantly, branch-specific facilitation required both cAMP response element binding protein (CREB)-mediated transcription and protein synthesis in the SN, despite the SN cell body being common to both MN synapses. Although CREB-dependent transcription is a uniquely somatic event, Martin and colleagues elegantly demonstrated that protein synthesis in SN neurites did not require the cell body: serotonin stimulation produced *de novo* protein synthesis in SN neurites regardless of a SN soma being present [11]. Moreover, when serotonin-mediated LTF was induced in one SN-MN synapse, a single exposure to serotonin, which is normally not capable of inducing LTF on its own, was able to induce LTF at the unstimulated synapse. Protein synthesis inhibitors had no effect when co-applied with a single pulse of serotonin on the second branch, but did block LTF at both branches when applied concurrently with the initial 5 pulses of serotonin on the first branch [11]. These findings highlight two critical principles: (i) there is a retrograde signal from the synapse to the soma to initiate transcription, and (ii) mRNAs can be stored locally at the synapse for rapid translation.

Taken together, these seminal experiments indicate there is an as yet undetermined contribution of the molecular signaling induced at activated synapses that also modulates the local ‘neighborhood’ of an activated synapse to facilitate more widespread activity- and location-dependent changes in neural responses. Viable candidate mechanisms that could regulate this phenomenon include the wide variety of kinases that become activated during learning and memory formation, including the MAPK and PKA families of kinases.

### 2.2. Protein kinases in memory formation: Mitogen-activated protein kinase (MAPK) family

The MAPK family of kinases is important for many cellular functions including proliferation, differentiation, gene expression, and mitosis, in addition to playing a crucial role in learning and memory formation [12–14]. One member of the MAPK family, ERK1/2, can be considered a molecular ‘node,’ as many upstream pathways can contribute to, and many downstream consequences result from its activity [15]. The activation of ERK1/2 itself is mediated by small G-proteins, which respond to a variety of cell stimulation, including membrane depolarization and calcium influx, both of which are fundamental to neuronal communication and plasticity, to initiate a cascade of phosphorylation events (MAPKK kinase → MAPK kinase → MAPK) [16,17].

The same upstream mediators responsible for activating ERK1/2 can additionally activate other members of the MAPK family, including c-Jun N-terminal kinases (JNKs) and p38 MAPK [12,18,19]. The JNKs are stress-activated proteins that are regulated by as many as 13 MAPKKKs, and, similarly to ERK1/2, many different stimuli may affect their activation [19,20]. JNKs phosphorylate the transcription factor c-Jun, which then binds to DNA and helps to initiate transcription [21]. c-Jun can associate with another transcription factor, c-Fos, to form the AP-1 transcriptional complex, which can in turn regulate the expression of several genes including those related to cell cycle progression and differentiation [19]. Intriguingly, AP-1 transcription factors are critical for positive feedback loops induced by brain derived neurotrophic factor (BDNF) in cortical neurons [22], a growth factor critical for long-lasting memory and plasticity (reviewed below). Moreover, AP-1 DNA binding activity is increased after LTP induction in the dentate gyrus [23], and a specific inhibitor of JNK phosphorylation, but not ERK1/2 or p38 MAPK phosphorylation, enhanced STM and blocked LTM for inhibitory avoidance in rats [24].

p38 MAPK is a particularly interesting member of the MAPK family, as its activation is required for the expression of long-term depression (long-lasting synaptic weakening; LTD) in *Aplysia* [25]. Upon activation by the inhibitory neuropeptide FMRFa, p38 MAPK phosphorylates CREB-2, which binds to the promoter region of the immediate early gene and transcription factor, C/EBP, preventing its transcription [25]. This is of particular consequence because C/EBP, like the MAPKs, is an evolutionarily conserved molecule required for learning and memory formation in vertebrates and invertebrates [26–28]. Indeed, inhibiting p38 MAPK activity blocks LTD and facilitates LTF, while its specific activation facilitates LTD and blocks LTF [25]. In the dentate gyrus of rats, a specific inhibitor of p38 MAPK similarly attenuated LTD, but had no effect on LTP [29]. Moreover, activation of p38 MAPK in combination with tyrosine de-phosphorylation was sufficient to induce LTD in the CA1 region of the hippocampus [30]. This is in direct opposition to the role of ERK1/2 in the induction of plasticity and memory, though as in the case of many different kinases, there may be interactions between p38 MAPK and ERK1/2 in LTD (see [31] for review). While ERK1/2 is directly activated by MEK, a MAPKK, p38 MAPK is directly activated by MKK3 and MKK6, all of which are regulated by the small G-protein Ras [32]. Because Ras activity can in theory result in the concurrent activation of p38 MAPK and ERK1/2, as is demonstrated in rat cortical neuron culture and *Aplysia* SNs [33], this entwined activation scheme produces the potential for different members of the MAPK family to act concurrently as integrative counterparts. This notion is important to consider when predicting the functional outcomes of parallel MAPK cascade activation, including transcriptional regulation as briefly discussed here.

Of particular interest in understanding the role of a kinase family as ubiquitous and complex as the MAPK system is its kinetic properties and temporal activation patterns in response to learning-related stimuli. ERK1/2 is activated in different temporal phases, both during and after training paradigms that induce long-term synaptic and behavioral plasticity [34–43]. Importantly, each temporal phase of ERK1/2 activity can be of critical consequence to LTM induction and maintenance. In *Drosophila*, Pagani and colleagues [39] reported that in response to an associative odor-shock training trial, ERK1/2 is activated at approximately the time point which corresponds to the permissive inter-trial interval (ITI) used to induce associative LTM (similar observations are reported in [36,43]). When the next training trial was delivered, ERK1/2 activation decreased to baseline, and then gradually increased again to a maximal level that coincided with when the subsequent training trial in the pattern was to be delivered [39]. Interestingly, in a model of Noonan’s syndrome in which the protein tyrosine phosphatase SHP2 is mutated, the temporal profile of ERK1/2 activation in response to a training trial is prolonged, and the pattern of repeated-trial training permissive for LTM formation in wildtype flies results in impaired LTM in SHP2 mutant flies. Pagani and colleagues [39] were able to correct this deficit simply by increasing the ITI to align with the altered kinetics of ERK1/2 activation and de-activation. Moreover, models of the temporal kinetics of kinase activation, including ERK1/2, can successfully predict non-intuitive training patterns which result in robust LTF in *Aplysia* [44]. These data reveal two important concepts: (i) ERK1/2 activation is important for binding multiple training trials into a unified long-lasting memory, and (ii) the temporal kinetics of kinase activation may confer ‘coincidence detection’ to the cell, a notion that we will discuss further below.

In addition to a complex temporal profile, ERK1/2 has a highly complex subcellular spatial activation pattern, and can be activated both at the synapse as well as at the soma to exert distinct effects unique to each compartment (e.g. receptor phosphorylation and gene expression, respectively) [45]. There is an additional level of spatial regulation that is not well appreciated in the context of learning and memory: small G-protein and scaffold localization (see [46] for review). Casar and colleagues [47] experimentally manipulated localization of the small G-protein Ras via constructs known to specifically localize to the endoplasmic reticulum (ER), lipid rafts, the disordered plasma membrane, or the Golgi complex, in HEK293T cell cultures. When ERK1/2 is activated via Ras localized to lipid rafts, but not the ER of Golgi membranes, it phosphorylates cytoplasmic/transmembrane proteins such as epidermal growth factor receptor and cytoplasmic phospholipase A2. However, when Ras was localized to the disordered plasma membrane, ERK1/2 activation resulted in phosphorylation of RSK1, a CREB kinase that shuttles between the cytoplasm and nucleus. The activation of the transcription factor Elk1, which can be phosphorylated by ERK1/2, p38 MAPK, and JNK1/2, was diminished by the inhibition of ERK1/2 regardless of the localization of Ras, though less so if ERK1/2 was activated by Ras at the Golgi membrane. Conversely, if JNK1/2 was inhibited, the most pronounced effect on Elk1 activation resulted when Ras was localized to the Golgi membrane. Casar and colleagues [47] further demonstrated that these effects resulted from the utilization of different scaffolding molecules, such as kinase suppressor of ras1 (KSR1), which is required for LTP and associative learning [48]. Interestingly, the small G-proteins Raf1 and B-Raf are widely distributed throughout the rat brain, but occupy slightly different subcellular compartments: Raf1 is primarily cytosolic surrounding the nucleus, while B-Raf is widely distributed throughout the cell [49]. These data collectively suggest that seemingly small changes in the location or mechanism by which MAPKs are activated during learning-related stimuli can in principle result in distinct routing of MAPK activation, which in turn could confer a unique message to the cell.

### 2.3. Protein kinases in memory formation: cAMP-dependent protein kinase (PKA)

PKA is a serine/threonine kinase that is activated by intracellular cAMP produced by adenylyl cyclase in response to cell stimulation. PKA has four subunits: two regulatory subunits that tonically inhibit two catalytic subunits. Binding of cAMP to the regulatory subunits results in the dissociation of the catalytic subunits, which are then free to phosphorylate their substrates in the cytosol and nucleus [50–52]. PKA activity has been demonstrated to be required for synaptic plasticity and memory formation in both invertebrates [53,54] and vertebrates [51,55]. Interestingly, the persistent activation of PKA was shown to be critical for 12 hours after the induction of LTF in SN-MN synapses in *Aplysia*[56], which is particularly remarkable given that significant PKA activation is measured in SNs within 5 minutes of a single training trial [57]. Indeed, prolonged stimulation that leads to transcription-dependent LTF and LTM in *Aplysia* results in a more persistent increase in cAMP levels and PKA activation, causing the catalytic subunit of PKA to translocate into the nucleus where it phosphorylates transcription factors important for inducing new gene expression required for long-term plasticity, including CREB-1 [58–60].

Surprisingly, Pu and colleagues [61] reported that chronic exposure (10 days) to opiates impairs both hippocampal LTP and LTM, which was (i) correlated with increased hippocampal PKA activity and (ii) reversed by administration of PKA inhibitors. These data suggest that over-activation of PKA can disrupt LTP induction, stressing the need for appropriate temporal regulation of PKA activity in learning-related plasticity in the hippocampus. Moreover, similar to that of ERK1/2, the temporal profile of PKA activity may encode coincident learning experiences. Mice subjected to strong contextual fear conditioning training consisting of 3 trials with a one minute ITI exhibited LTM that was sensitive to PKA disruption immediately after training, but not one or more hours after training [62]. However, mice subjected to weak contextual fear conditioning training consisting of a single trial exhibited LTM that was sensitive to PKA disruption immediately and 4 hours after training, but not 1, 6, 8, or 23.5 hours after training [62], suggesting that a single training trial induced distinct phases of PKA signaling, while multiple training trials did not.

PKA activation is also spatially regulated by scaffolding molecules, most notably A Kinase anchoring proteins (AKAPs) [63,64]. In fact, Huang and colleagues [65] suggest that AKAP anchoring of PKA may mediate synaptic tagging and capture. A competitive inhibitor of AKAP-PKA interactions, stHt31, impaired late-LTP in the CA1 region of mouse hippocampal sections induced by electrical stimulation of Schaffer collaterals without impairing the induction of chemically-induced LTP via global increases in cAMP. These data suggest that LTP induction via pathway stimulation that results in synapse-specific potentiation requires the accurate localization of PKA by AKAPs. Indeed, strong stimulation of one region of the Schaffer collaterals-CA1 pathway was no longer capable of facilitating the induction of late-LTP in a neighboring region when weak stimulation was delivered one hour later [65], suggesting that the molecular processes that induce synaptic tagging and capture (discussed above) are disrupted if PKA is inappropriately localized.

### 2.4. Interactions between distinct protein kinases in memory formation

Taken together, the data reflecting exquisitely precise spatial and temporal properties of molecular signaling during memory formation indicate that there is a complex and diverse network of molecular cascades induced in parallel in response to learning events. However, when each molecular event is studied in isolation, any interactions and/or synergism among these simultaneously engaged signaling cascades is difficult to determine. An example of this notion is the relationship between MAPK and PKA in transcriptional regulation in *Aplysia*.

Transcription can be initiated by several mechanisms, one of which is the activation of the transcription factor CREB. In *Aplysia*, the induction of gene expression by the isoform of CREB that activates transcription, CREB-1, can be endogenously repressed via the DNA binding capability of CREB-2 [66], suggesting that there may be two different pathways to regulate the initiation of CREB-mediated transcription: via activation and de-repression. Indeed, in *Aplysia*, PKA directly phosphorylates CREB-1, thereby activating it [58,59], while MAPK phosphorylates CREB-2, resulting in de-repression [67,68]. There are reports in mammalian studies of a similar mechanism by which the CREB family both activates and represses transcription. While both the mammalian homolog of CREB-2, activating transcription factor 4 (ATF4), as well as a homodimers of a related isoform, ATF3, have been reported to repress transcription (see [69] for review), ATF4 may act as a passive repressor of the transcription of certain genes while mediating differential gene expression itself [70]. Indeed, while ATF4 is upregulated in the brains of patients with and mouse models of Alzheimer’s Disease, suggesting a correlation between memory dysfunction and increased transcriptional repression, transgenic mice without ATF4 have impaired LTP, LTD, and memory [71], indicating that the relationship between CREB isoforms in mammals may be more complicated. Interestingly, ATF4 associates with Disrupted-In-Schizophrenia 1 (DISC1) and together bind to the phosphodiesterase 4D gene and repress its expression [72], suggesting that the binding partners with which ATF4 associates may in part dictate its role in transcriptional regulation.

In addition to the synergism between MAPK and PKA in regulating transcription, studies in both hippocampal and *Aplysia* neurons showed that PKA phosphorylation of CREB-1 requires MAPK activation [73], while nuclear translocation of MAPK requires PKA activation [74,75]. A similar inter-dependency between the consequences of MAPK and PKA signaling have been demonstrated both during LTP in the CA1 region of the hippocampus [76], LTM for inhibitory avoidance in rats [77] and for intermediate-term facilitation in *Aplysia* [42].

These data reviewed here collectively highlight the inter-dependent roles of distinct kinases in connecting extracellular signals to transcriptional regulation and ultimately memory formation. Furthermore, the remarkably tightly controlled temporal and spatial regulation of MAPK and PKA activity suggest that molecular signaling cascades engaged by learning-related stimuli must occur in a temporally and spatially coordinated manner to successfully support long-lasting plasticity and memory. While reviewing MAPK and PKA activity is a platform on which to begin to explore the consequences of spatial and temporal molecular networks, there are many other signaling cascades with elaborate activity profiles that are critical for learning and memory formation. These include the Ca^2+^/calmodulin-dependent protein kinase (CAMK) family [78,79], PKC and the persistently active atypical isoform of PKC, PKMζ [80,81], as well as mTOR-mediated protein synthesis [82,83,84,85]. Though an exhaustive evaluation of each molecular cascade is outside the scope of this review, it is important to note that several models of plasticity predict complex dynamics within and between these signaling families [44,86–88].

What remains unclear, however, is how precise spatial and temporal molecular networks, and complex interactions between intracellular signaling cascades, is ultimately initiated. Because virtually all kinases can be activated via several upstream mechanisms and second messenger systems, one hypothesis is that the extracellular signals that orchestrate intracellular signaling (i.e. neurotransmitters, hormones, neuromodulators, etc.) are themselves spatially and temporally regulated in distinct molecular networks. Growth factor (GF) signaling, which may very well mediate many of the events described above, is an excellent platform on which to begin to synthesize the data into a coherent, dynamic molecular network. We will take up this idea in the section that follows.

## 3. Growth Factor Signaling Networks in Learning and Memory

GF signaling cascades are tightly regulated in both space and time during developmental plasticity in order to create precise neural circuits [89–91], and these same principles are at play during GF regulation of learning and memory formation [92]. GF signaling thus affords an ideal opportunity to demonstrate the concept of temporal and spatial networks in learning and memory.

GFs are secreted molecules that bind membrane-associated extracellular receptors to initiate intracellular signaling cascades. Since the first fully characterized GF, nerve growth factor (NGF; [93,94]), it has become apparent that there are several families of GFs with unique receptors, the two major classes of which are receptor tyrosine kinases and serine-threonine kinases. Importantly, the high level of interaction between intracellular signaling cascades, including the kinases reviewed here, would predict that interactions also occur between multiple upstream signaling activators, like GFs. Indeed, a wide variety of GFs are engaged in learning and memory formation, and no GF by itself can mediate the complex molecular and cellular outcomes critical for long-lasting plasticity, (for a review on GF interactions mediating dendritic plasticity, see [92]). We thus propose that characterizing the parallel engagement of unique families of GFs supporting learning and memory formation can yield a deeper understanding of the molecular signaling networks involved in this long-lasting and dynamic plasticity.

### 3.1. Spatial and temporal GF signaling networks in learning and memory formation

GFs are capable of playing different roles in the different cellular and subcellular compartments in which they are engaged. In particular, mammalian neurotrophins, including NGF, BDNF, NT-3, and NT-4/5, have been extensively studied as signaling modulators critically involved in synapses undergoing learning-induced plasticity. Indeed, numerous studies have shown that the mRNAs and proteins of neurotrophins are widely distributed throughout the mammalian CNS in distinct, but largely overlapping expression patterns in regions important for learning and memory including the hippocampal formation, amygdala, and cortex [95–97]. Additionally, some studies suggest that neurotrophins can exist within different subcellular compartments within these brain regions, including axonal localization [98–103] and somatodendritic localization [98,104–107]. Furthermore, both anterograde [98,99,103] and retrograde transport [104,108] of neurotrophins has been reported, suggesting that the site of GF initiation may not be the same or only site of GF action. This can occur through endocytosis of the GF-GF receptor complex upon ligand binding, and thus may be an important means of synapse-soma communication. In addition to neurotrophins forming intracellular signaling complexes, or endosomes [109], both epidermal growth factor [110] and insulin-like growth factor II (IGF-II; [111,112]) can be regulated in a similar manner. Although not well studied in the context of learning and memory, kinase phosphorylation has been demonstrated to be a critical regulator of internalized GF receptors into either a recycled population or a degraded population [113]. Because many GF receptors are both upstream activators for kinases as well as substrates for their modulation, this sort of mechanism may represent an as yet unappreciated regulation of both spatial and temporal molecular signaling.

Adding to the complexity, neurotrophins can be secreted from either postsynaptic dendritic spines or from presynaptic terminals, and both their synthesis and release in the adult central nervous system seems to be activity-dependent [95,97,114,115]. Indeed, membrane depolarization in hippocampal cultures via high potassium solutions or glutamate treatment increases mRNA expression of BDNF and NGF in addition to increasing the release of BDNF, NGF, and NT-3 [116–120]. Moreover, the induction of GF mRNAs in hippocampal slices appears to be spatially restricted to the area of stimulation in response to LTP-inducing stimuli: Patterson and colleagues [121] reported that stimulation of the CA1 region leads to an increase in mRNA levels of both BDNF and NT-3 in CA1, but not in neurons in the CA3 or dentate gyrus regions. Interestingly, in addition to activity-dependent release, there is also evidence for neurotrophin-induced neurotrophin secretion [122,123], which raises the interesting question regarding the functional significance of activity-dependent synthesis and release of multiple neurotrophins at the same synapse. Autocrine or paracrine actions of released neurotrophins have been reported [95,96,124], however the simultaneous actions of multiple growth factors released at the same synapse have yet to be explored. Moreover, it will be important to examine and dissect the parallel or convergent signaling cascades downstream of GF receptor activation within the same cell.

Once released at a synapse, neurotrophins as well as other GFs bind to their putative receptors and induce intracellular signaling leading to changes in synaptic transmission and connectivity. Multiple studies in hippocampal and cortical cultures and slices [125,126] have reported that treatment with each of the neurotrophins (BDNF, NGF, NT-3, and NT-4/5) induces potentiation of excitatory glutamatergic synaptic transmission through either presynaptic [127,128] and/or postsynaptic mechanisms [129,130]. Interestingly, in addition to facilitation of excitatory synaptic transmission, there have also been reports of neurotrophin-mediated depression of inhibitory synaptic transmission [125,131], which would result in a similar functional outcome (increased excitability) albeit through a very different molecular cascade (decreased inhibition).

Activity-dependent release of GFs, which is common during a learning event, often occurs at the synapse [97]; however, GFs can mediate both synaptic and somatic events. For example, BDNF signaling induced by IA training in rats regulates the activity of several proteins important for LTM formation at the synapse, such as CAMKIIα, ERK1/2, and AKT, as well as CREB phosphorylation, which is a specifically nuclear, and thus somatic, protein [132]. Moreover, Kanhema and colleagues [133] demonstrated that an infusion of BDNF into the dentate gyrus of anesthetized rats induced LTP, and increased phosphorylation of translational machinery proteins in a spatially segregated manner. Specifically, BDNF increased eIF4E phosphorylation in synaptically-enriched samples, and increased eEF2 phosphorylation in global, but not synaptically-enriched samples. Interestingly, both effects were MEK-dependent, suggesting that BDNF signaling can make use of the same signaling broker, perhaps in different cellular compartments, for different outcomes.

GF signaling is also engaged in temporally discrete profiles during learning and memory formation. BDNF signaling, as discussed above, is often engaged very early in response to neuronal activity [97]. Interestingly however, in general GFs are recruited in a delayed fashion well after a learning event or learning-related stimulus. For example, IGF-II mRNA and protein is increased in the dorsal hippocampus 20 hours, but not 6 or 9 hours after inhibitory avoidance (IA) training in rats, and its signaling via the IGF-II receptor is required for more than 1, but less than 4 days, after IA training for memory consolidation [134]. Moreover, NGF levels in the hippocampal CA1 region were increased one week after contextual fear conditioning in rats, but not earlier or later [135], and inhibiting NGF signaling 1 week, but not 4 weeks, after training impaired memory [135]. Activin, a member of the TGFβ superfamily, was required for specifically late-LTP several hours after high frequency stimulation [136], and LTP could be enhanced by exogenous activin application or blocked by activin inhibitors 1 hour, but not 3 hours after induction [136]. There is even evidence that BDNF is additionally required long after training to support memory persistence. Blocking protein synthesis in the hippocampus 12 hours after IA training disrupts the expression of memory 7 days, but not 2 days, after training [137,138], which can be rescued by co-injection of BDNF [138]. Moreover, Bambah-Mukku and colleagues [124] have recently demonstrated a BDNF-induced positive feedback loop that supports long-term IA memory in rats. IA training induces BDNF-dependent CREB activation and C/EBPβ expression; C/EBPβ then mediates an increase in BDNF gene and protein expression, which constitutes an additional phase of BDNF signaling required for LTM formation.

Despite extensive data reporting very precise spatial and temporal regulation of both intracellular signaling (i.e. kinase activation) as well as their upstream activators (i.e. GFs), a critical issue remains: what is the meaning of this signaling for the cell? Specifically, how are these disparate (both temporally and spatially) molecular networks integrated into a coherent message? And, what is the value of this complexity? One possible answer is that the integration of individual spatiotemporal profiles is capable of relaying vastly more information than each signal considered in isolation. It is thus possible that individual temporal and spatial molecular networks interact to serve “coincidence detection” functions. In the section that follows we will examine possible mechanisms of spatial and temporal integration, specifically highlighting mechanisms of synapse—soma communication as this trans-compartmental communication contains both a temporal and spatial component. We will then review in detail a specific example of the notion of coincidence detection in GF signaling networks: Two-Trial LTM formation in *Aplysia*.

## 4. Cellular Interpretation of Spatial and Temporal Molecular Networks

### 4.1. Integration of distinct molecular networks: How do synapses and cell bodies communicate?

Long-term forms of cellular and behavioral plasticity are distinguished from other forms of memory (e.g. working, short-term, and intermediate-term memory) by their requirement of *de novo* gene expression [26,53]. However, there is abundant evidence that synapses are the initial site of plasticity, making the mechanisms by which large numbers of synapses on any one neuron communicate with a single cell body, and vice versa, of particular interest.

Microtubules are dynamic elements of the cell structure that serve as ‘tracks’ upon which molecular motors can transport cargo. Microtubules typically run throughout the cell and are able to integrate indirectly with the actin framework through cross-linking cytoskeletal proteins such as the CLASP family of proteins [139–141]. There are diverse families of microtubule motors, such as kinesins, for transporting molecular cargo in the anterograde direction (i.e. from soma to synapse), but not nearly the same diversity in retrograde (i.e. from synapse to soma) motor proteins, such as dyneins [142,143]. Additionally, the actin cytoskeleton is traversed by myosin proteins [142]. Both kinesins and myosins move across microtubules and actin, respectively, via phosphorylation-induced changes in protein conformation, whereas dyneins move in a more complex manner, with their head regions making contact with microtubules [142]. Moreover, while kinesins and myosins directly attach to vesicles via anchoring proteins [142], dyneins complex with cargo via sets of scaffold proteins, such as the dynactin protein complex, where vesicular cargo must be attached indirectly to the dynein protein [144]. The kinesins and dyneins also yield different temporal kinetics of transport. Kinesins have a high duty ratio, meaning that they can quickly transport vesicular cargo long distances (several micrometers) without falling off of microtubules, while dyneins have a low duty ratio [144], collectively resulting in much faster transport from soma to synapse than vice versa.

Microtubules tend not to extend into dendritic spines, the primary sites of synaptic input, where instead there is a high concentration of actin filaments [139,140]. Myosin-mediated transport on actin filaments is largely responsible for organelle translocation and relatively slow cellular movement [139], in contrast to kinesin-mediated transport on microtubules which is responsible for fast, axonal transport of small vesicular cargo [144]. Interestingly, microtubules have been reported to ‘invade’ dendritic spines in mouse hippocampus in response to NMDA receptor-dependent calcium influx, a phenomenon dependent on actin polymerization and the expression of Drebrin, a protein that facilitates the interaction between actin filaments and microtubules [145]. Importantly, microtubule content in dendritic spines is primarily observed when dendritic spines are active [145], suggesting that microtubule invasion into active dendritic spines could serve as an activity-dependent and transient trafficking route for vesicular cargo traveling between the soma and synapse. Indeed, increased vesicular trafficking as well as increased kinesin heavy chain mRNA have been reported in response to learning-related stimuli in *Aplysia* [146]. Together, these data raise the intriguing possibility that somatic gene and protein expression must be spatially and temporally coordinated in such a way as to take advantage of the transient availability of transport to activated synapses. Moreover, the molecular mechanisms which regulate these windows of transport may be of critical importance to the successful induction of long-lasting plasticity, and to our knowledge have not yet been addressed.

### 4.2. Integration of distinct molecular networks: A case study of coincidence detection in Aplysia

We have briefly discussed how the soma and synapse can communicate, and some aspects of the temporal regulation of this communication. However, because molecular cascades are often studied in isolation, it is experimentally difficult to determine if there are instances in which distinct molecular networks are integrated to mediate functional outcomes that neither network, on its own, can support. We will now review a study using the marine mollusk, *Aplysia*, as a model, which demonstrates the spatial and temporal integration of distinct GF signaling networks to synergistically support LTM formation.

The simple central nervous system of *Aplysia* is well-suited for interrogating the molecular mechanisms of learning and memory formation [53]. The defensive withdrawal reflexes are largely governed by monosynaptic sensory neuron (SN) communication with motor neurons (MNs) [147–149]. Upon training with electrical stimulation or the neuromodulator serotonin, the withdrawal reflexes become enhanced, or sensitized, and similar to vertebrates, LTM for sensitization in *Aplysia* lasts days to weeks and requires both translation and transcription [150].

Memory formation in *Aplysia*, like other species, is responsive to the pattern of training. For instance, four or five repeated, spaced training trials [151] yields superior LTM than the same amount of training given with little time in between training trials [43,152], which is a well-conserved phenomenon known as the spacing effect [153]. MAPK activation (homologous to ERK1/2) at SN cell bodies occurs in two different phases in response to sensitizing stimuli in *Aplysia*: (i) during the training interval and (ii) after training. A single training trial induces an early phase of MAPK activation in SN somata 45 minutes after Trial 1 [37,43]. This early phase is transient, and in the absence of additional training trials it decays to baseline by 60 minutes [37,43]. However, following repeated trial training that is permissive for the induction of LTM, MAPK is persistently activated in the SN somata 1–3 hours after training [35,42,154]. Importantly, both the early and late phases of MAPK activation are required for LTM formation [35,37].

Philips and colleagues [37,43,149] developed a novel LTM training paradigm, consisting of only two training trials spaced by 45 minutes (Two-Trial training), which is sufficient to induce LTM in *Aplysia*. Interestingly, Trial 2 must be delivered within a restricted temporal window: if occurring at 15 minutes or 60 minutes after Trial 1, no LTM is induced [37,43]. Collectively, data from Carew and colleagues suggest that Trial 1 induces a “molecular context” at 45 minutes that includes MAPK activation, its translocation into the nucleus of SNs, and increased gene expression of the immediate early gene *c/ebp* [37]. Trial 2 then interacts with this molecular context and causes changes which make the circuit permissive for long-lasting information storage. With this minimalistic training pattern, Two-Trial training affords the unique opportunity to assay molecular and cellular changes induced by each training trial individually, as well as to explore the interaction between the molecular context established by Trial 1 and the changes induced by Trial 2. Two GF families have been reported to activate MAPK and be required for long-term plasticity in *Aplysia*: neurotrophins signaling via the receptor TrkB [155,156] and the TGFβ superfamily signaling via the receptor TGFβr-II [154,157–160]. Thus, as a first step in characterizing the role of different GF families during LTM formation, Kopec and colleagues [161] analyzed the necessity for TrkB and TGFβr-II signaling upstream of the early and late phases of MAPK activation induced by Two-Trial training.

The data demonstrated a striking double-dissociation in the temporal and spatial regulation of these GFs during learning-related stimuli. Trial 1 initiated TrkB signaling at the SN-MN synapse, which was required for early-phase MAPK activation in SN cell bodies. Importantly, these data necessitate an intracellularly transported retrograde signaling to exert the somatic effects of synaptic TrkB signaling. Trial 2 initiated TGFβr-II signaling at SN cell bodies, which was required for late-phase MAPK activation in the same compartment. Surprisingly, the later, TGFβr-II-dependent phase of MAPK was not disrupted if Trial 1 TrkB signaling was inhibited, suggesting that these two GF families were signaling independently despite being in temporal sequence.

Kopec and colleagues [161] next sought to determine if these spatiotemporally regulated GF signaling cascades regulated a molecular event uniquely required for long-lasting plasticity and memory: transcription. C/EBP, briefly mentioned above, is an immediate early gene and transcription factor that is evolutionarily conserved and required for LTM formation [26]. Moreover, both Trial 1 and Trial 2 result in increased *c/ebp* mRNA expression in *Aplysia* [37,161]. A unique syngergistic interaction emerged when GF signaling upstream of *c/ebp* expression was examined. In agreement with the MAPK data, Trial 1 TrkB signaling was required for Trial 1 *c/ebp* expression, and Trial 2 TGFβr-II signaling was required for Trial 2 *c/ebp* expression. However, unlike the MAPK data, when TrkB signaling was inhibited during Trial 1, Trial 2 *c/ebp* expression was also disrupted. These data suggest that Trial 1 TrkB signaling increases the expression of *c/ebp* mRNAs (presumably through transcriptional activation, though this has not yet been tested), and Trial 2 TGFβr-II signaling, rather than engaging a new wave of transcription, prolongs the expression of Trial 1 mRNAs. Finally, in a set of experiments to determine if these molecular observations were behaviorally relevant, Kopec and colleagues [161] demonstrated that Trial 1 TrkB signaling and Trial 2 TGFβr-II signaling were, indeed, required for Two-Trial LTM for sensitization in *Aplysia*.

**Figure 1:**
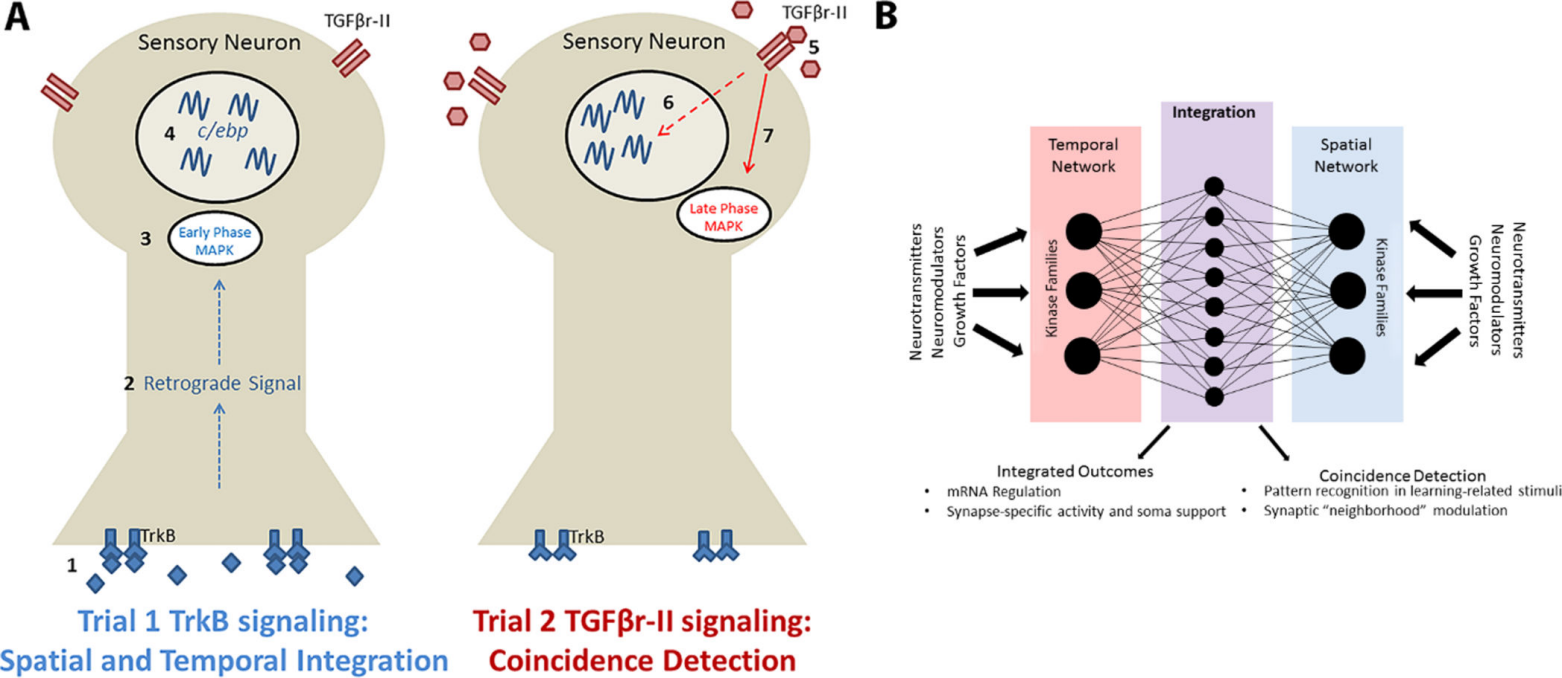
Neuronal interpretation of spatial and temporal molecular networks in long-lasting plasticity and memory **A.**Distinct GF signaling networks (TrkB and TGFβr-II) synergistically interact to mediate LTM formation in *Aplysia*. (1) Trial 1 induces release of TrkB ligands at the SN-MN synapse, which results in (2) an intracellularly transported retrograde signaling that is responsible for (3) a transient and delayed phase of MAPK activation in SN somata. Concurrently, TrkB signaling mediates (4) an increase in expression of the immediate early gene, *c/ebp*. The delivery of Trial 2 during this molecular context induces (5) TGFβr-II ligand release at SN somata. TGFβr-II signaling then interacts with the TrkB signaling cascade to (6) prolong the expression of *c/ebp* mRNA established by Trial 1, and (7) independently mediates a persistent phase of MAPK activation. These molecular observations demonstrate both the spatial and temporal integration of distinct molecular networks as well as one possible role this complexity serves: coincidence detection. **B.** Reports reviewed here indicate that there are exceedingly complex spatial and temporal profiles of both extracellular (e.g. GFs) and intracellular (e.g. kinases) molecular signaling cascades. We propose that the integration of these distinct molecular networks serves to provide the neuron with more information than any one network can provide alone (e.g. coincidence detection). Thus, the initiation of complex molecular programs, including those underlying long-lasting cellular and behavioral plasticity, is best understood as synergistic interactions between multiple signaling networks.

This report exemplifies many of the principles discussed in the present review (see Figure 1A). First, there are spatially and temporally distinct molecular networks, specifically GF signaling networks: TrkB signaling was initiated synaptically to mediate early, Trial-1 dependent molecular outcomes (i.e. MAPK activation and *c/ebp* expression). Conversely, TGFβr-II signaling was initiated somatically to mediate later, Trial-2 dependent molecular outcomes. Moreover, TrkB signaling resulted in synapse-soma communication, while TGFβr-II signaling prolonged the expression of existing mRNAs. Importantly, Trial 1, despite activating TrkB signaling, synapse-soma communication, MAPK, and transcription, cannot by itself produce LTM [43]. Transcription is a complex, highly integrative cellular function, requiring the collaborative activity of several different families of proteins and protein complexes. The fact that *de novo* gene expression readily occurs in response to stimuli that alone cannot induce LTM is of critical importance: transcription is necessary, but not sufficient, for the formation long-lasting plasticity. Instead, the coincident arrival of another learning-related signaling cascade, at the appropriate time and in the appropriate subcellular compartment, must occur. Thus, the molecular events induced by Trial 2 serve a coincidence detection role in the cell such that the circuit initiates long-lasting information storage.

Precisely what signal induced by Trial 2 is most salient at conferring coincidence is at present unclear, and it is likely that an interaction of several events contribute to this process. However, because TGFβr-II signaling during Trial 2 is required for Two-Trial LTM formation, consequences of the signaling via this GF are likely to be involved. These include, but are not limited to (i) an independent phase of MAPK activity, which could have numerous downstream outcomes, and (ii) prolonged *c/ebp* expression, which could result in the increased likelihood of its translation, and thus increased activity of its functions as a transcription factor (i.e. more new gene expression). Interestingly, *c/ebp* mRNAs contain an AU-rich element in their 3’ untranslated regions, which is a motif targeted by the Hu/ELAV family of RNA binding proteins that can stabilize mRNAs [162–164]. Post-transcriptional regulation by RNA binding proteins has been proposed to serve as a method by which the cell filters out transcriptional noise and ensures the degradation of contextually irrelevant transcripts [165], and thus RNA binding proteins are one way in which newly transcribed mRNAs may be regulated. The phosphorylation state of Hu, which has been reported to be modulated by PKCδ, p38 MAPK, and AMP kinase dictates its ability to bind to target mRNAs and shuttle them between the nucleus and the cytoplasm, which in turn regulates the probability that mRNAs associate with ribosomes for translation [166–168]. Thus post-transcriptional regulation of mRNAs could be a site of integration for the signaling of several different kinases and potentially GF signaling cascades. If TGFβr-II signaling does, indeed, recruit RNA binding proteins, other mRNAs not yet reported to be upregulated by Trial 1 may additionally be stabilized.

## 5. Consequences of Spatial and Temporal Molecular Networks: Experimental and Therapeutic Implications

Collectively, the studies reviewed here support the notion depicted in Fig. 1B: neurons integrate spatially- and temporally-regulated molecular signaling networks, and in response, initiate complex molecular programs required for long-lasting cellular and behavioral plasticity. Moreover, this integration provides more information than any one signaling network alone, such as coincidence detection. Importantly, two recent publications utilizing transcriptomic profiling in development support this notion and demonstrate unexpected instances of interaction and integration [169,170]. The task of characterizing the temporal and spatial profiles of a molecular network is itself a difficult undertaking. A scenario involving two or more GFs (or other signaling brokers) would significantly increase the number of possible combinatorial effects, making these studies inherently difficult. Monosynaptic connections such as those underlying the defensive withdrawal reflexes in *Aplysia* offer the spatial resolution needed for these mechanistically detailed studies, as exemplified in the case study above. As it stands, five different GFs have been reported to have a role in plasticity in *Aplysia* [156,160,171–173], and there are many more GFs implicated in mammalian plasticity [92].

The notion of spatial and temporal molecular networks is particularly important from the perspective of translational research and therapeutic intervention. Many GFs, including BDNF-TrkB and TGFβ-TGFβr signaling, have been suggested to be dysregulated during cognitive dysfunction of various etiologies in humans, including Alzheimer’s disease [174–176]. GF receptors are ideal candidates for therapeutic interventions because their ligand binding domain is extracellular. However, the results reported by Kopec and colleagues [161] reviewed above emphasize three reasons why a therapy that targets a single GF family may be insufficient to ameliorate cognitive deficits. First, the targeted GF family may be only one of several GFs in a molecular network disrupted by pathology. Thus, restoring signaling from one GF family may restore only a single component of the signaling required for behavior. Furthermore, the targeted GF family may require the interaction with other GFs to fully achieve the functional outcomes of its molecular signaling. For instance, in the case of Two-Trial LTM in *Aplysia*, augmented or restored TrkB signaling may not be sufficient to restore LTM if TGFβr-II signaling is not efficiently interacting with TrkB-dependent signaling. Second, GF signaling is highly spatially regulated and this specificity may well be important for the neural circuit to correctly and effectively interpret the molecular signaling. Utilizing a systemic drug, despite having restored GF signaling, may disrupt the spatial signature of a GF family and thus disrupt behavior. Taking the data from Kopec and colleagues [161] as an illustrative example, if TrkB signaling was blocked at the synapse during Trial 1, but at the same time augmented at the soma, would this result in successful Two-Trial LTM formation? The delayed nature of the synaptic TrkB-dependent signal positions it to effectively interact with TGFβr-II signaling during Trial 2, and this may not be the result if TrkB signaling is instead engaged at the soma during Trial 1. And third, GF signaling also follows a strict temporal profile during LTM formation in *Aplysia*, which may well be the case for other forms of plasticity underlying various forms of learning. Without an understanding of *when* a GF family is recruited to support behavior, therapeutic interventions may miss the time frame in which it could be most effective. For instance, if TGFβr-II signaling was augmented one hour after Trial 1, *apc/ebp* expression would have returned to baseline levels and TGFβr-II signaling would not have the opportunity to interact with the TrkB-dependent signaling cascade, and thus may not result in the same behavioral outcome.

In conclusion, plasticity underlying memory formation is an exquisitely complex interplay between differential temporal and spatial molecular networks. Different signaling cascades, including kinases and GF signaling reviewed here, could be initiated in (i) an autocrine or paracrine fashion, (ii) simultaneously or in different temporal phases, resulting in (iii) local or long-distance (e.g. synaptic vs. somatic) molecular consequences, all of which increase the likelihood of interactions and synergism between cascades. Moreover, there can be salient information in these interactions and instances of synergy, such that cells can interpret ‘coincidence,’ pattern, and statistical probability in a series of learning-related stimuli. Discovering how cells integrate and interpret these dynamic networks is an extraordinary challenge, but this challenge is essential to tackle, as it is critical not only for understanding how the plasticity underlying learning and memory formation normally occurs, but also for designing effective therapies to intervene when this plasticity goes awry.
